# The Frank-Starling relationship of the heart revealed in a large animal study utilizing real-time undersampled radial MRI at variable inotropic state and heart rate

**DOI:** 10.1186/1532-429X-16-S1-P57

**Published:** 2014-01-16

**Authors:** Walter R Witschey, Francisco Contijoch, Jeremy R McGarvey, Victor A Ferrari, Michael Hansen, Julio Chirinos, Paul Yushkevich, Joseph H Gorman, Robert C Gorman, James J Pilla

**Affiliations:** 1 Surgery, University of Pennsylvania, Philadelphia, Pennsylvania, USA; 2 National Institutes of Health, Bethesda, Maryland, USA

## Background

LV performance is coupled to loading conditions (i.e. preload and afterload) and myocardial contractility (1,2). Therefore load-independence is an important means to isolate and study LV contractility in preclinical studies of cardiovascular pharmaceuticals and devices (3). Precise and rapid assessment of LV pressure is achieved with high fidelity catheter-based pressure transducers but measurements of LV volume are more difficult and less reliable. Thus far, MRI has not demonstrated sufficient spatiotemporal resolution to measure beat-to-beat changes of the pressure-volume (PV) loop to measure left ventricular (LV) elasticity or preload-recruitable stroke work (PRSW). Our aim was to develop a real-time (rtMRI) approach to measure PV and Frank-Starling relationships in a preclinical model of heart disease under normal and stressed physiologic conditions.

## Methods

The University of Pennsylvania IACUC approved all experiments. Yorkshire swine (n = 8) were anesthetized and instrumented with expandable vascular occluders placed at the inferior (20 mm) and superior (24 mm) vena cavae and transiently inflated during simultaneous rtMRI and intraventricular pressure measurement. Images were reconstructed with non-Cartesian parallel SENSE and >10 k frames were segmented in < 5 min with semi-automatic active contours.

## Results

**Figure 1:**
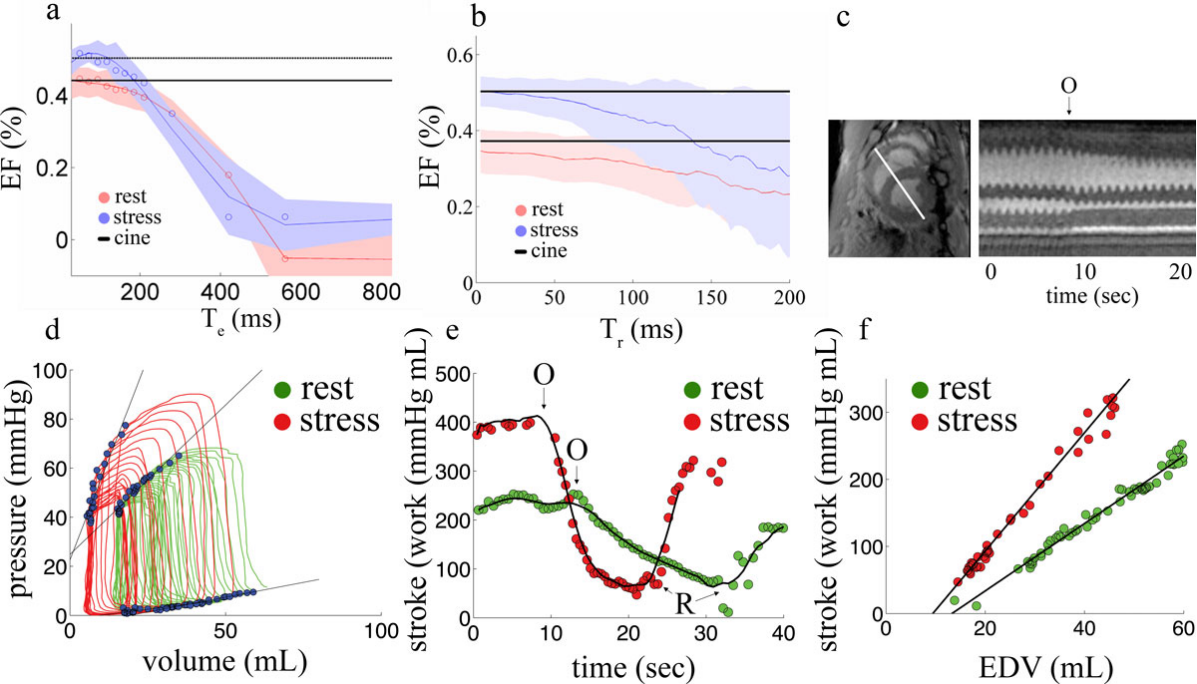
**rtMRI evaluation of PV loops and Frank-Starling relationship of the heart**. (a) Dependence of measured ejection fraction on image exposure time Te at rest and under continuous dobutamine infusion (stress). Rest and stress cine are shown with solid black lines. (b) Dependence of measured ejection fraction on image frame rate Tr at rest and stress. Rest and stress cine are shown with solid black lines. (c) cine MRI image (left) with solid white line indicating a projection through the rtMRI images (right). The start of inflow occlusion is labeled O. (d) Pressure-volume loops at rest and stress conditions. End-systolic and end-diastolic points are labeled (blue circles) and end-systolic and end-diastolic pressure-volume relationships are show with solid black lines. (e) Stroke work immediately prior to occlusion, during inflow occlusion and after release. (f) Dependence of stroke work on EDV (Frank-Starling relationship) for rest and stress conditions.

The relationship between Te, Tr and ejection fraction is shown (Figure 1a, b). We found PV loops could be accurately measured using an image exposure time Te < 100 ms (< 34 projections) and frame rate Tr < 50 ms (view sharing < 17 projections) at elevated heart rates (~140 bpm). With an optimized exposure time (Te = 95 ms and frame rate Tr = 2.8 ms), we found that there was no significant difference between cine and rtMRI at rest in end-diastolic volume (EDV), end-systolic volume (ESV), ejection fraction (EF), stroke volume (SV) or cardiac output (CO) (n = 8, p < 0.05) at either normal or elevated heart rates (table 1). A representative PV loop at rest and elevated inotropy is shown (Figure 1d). EES increased from 1.9 ± 0.7 to 3.1 ± 0.3 mmHg/mL (n = 8, p < 0.05). There was decreased stroke work (SW) under reduced loading conditions (Frank-Starling mechanism); PRSW was highly linear (r = 0.98) and increased from 6.2 ± 1.2 to 9.1 ± 0.9 mmHg during continuous IV dobutamine (n = 8, p < 0.05). There was no significant difference in the LV volume at zero pressure V0 (n = 8, p < 0.05).

**Table 1:** Real-time and cine-MRI derived LV hemodynamic and elastic properties

	Real-Time MRI (rest) (n = 5)	Real-Time MRI (stress) (n = 5)	Cine MRI (rest) (n = 5)	Cine MRI (stress) (n = 5)
Heart rate (bpm)	112.1 +- 22.9*	140.2 +- 20.2*	116.8 +- 21.1	147.6 +- 16.1

EDV (mL)	57.9 +- 13.2	47.0 +- 5.2	61.1 +- 10.7	54.1+-5.7

ESV (mL)	35.3 +- 7.1*	23.6 +- 4.1 *	35.8 +- 8.0	25.3 +- 10.7

SV (mL)	22.7 +- 8.3	23.4 +- 7.5	25.3 +- 5.8	28.9 +- 3.7

EF (%)	38 +- 9*	49 +- 11*	41 +- 7	53 +- 3

CO (L/min)	2.6 +- 0.7	3.2 +- 0.9	2.9 +- 0.6	3.3 +- 1.0

Eed (mmHg/mL)	0.3 +- 0.13	0.28 +- 0.19	N/A	N/A

Ees (mmHg/mL)	1.9 +- 0.7*	3.1 +- 0.3*	N/A	N/A

V0 (mL)	-8.1 +- 13.8	-1.9 +- 4.1	N/A	N/A

Stroke work (mmHg mL)	221.3 +- 51.9*	306.9 +- 96.4*	N/A	N/A

PRSW (mmHg)	6.2 +- 1.2	9.1 +- 0.9	N/A	N/A

* p < 0.05 paired t-test, N/A (not applicable) cine measures of elasticity and work were not possible to measure/not measured.

## Conclusions

RtMRI can accurately assess LV volumes, elasticity and PRSW at baseline and elevated inotropic state, providing non-invasive into the Frank-Starling relationship of the intact heart.

## Funding

The authors gratefully acknowledge support from the National Institutes of Health through awards K99HL108157 and R01HL63904.

